# Interictal short-term high-frequency cortical stimulation modulates epileptogenic zone and distributed network

**DOI:** 10.1093/braincomms/fcaf378

**Published:** 2025-09-30

**Authors:** Hao Yan, Ying Gao, Xueyuan Wang, Wei Shu, Liankun Ren, Tao Yu

**Affiliations:** Beijing Institute of Functional Neurosurgery, Department of Neurosurgery, Xuanwu Hospital Capital Medical University, Beijing 100053, China; Beijing Institute of Functional Neurosurgery, Department of Neurosurgery, Xuanwu Hospital Capital Medical University, Beijing 100053, China; Beijing Institute of Functional Neurosurgery, Department of Neurosurgery, Xuanwu Hospital Capital Medical University, Beijing 100053, China; Beijing Institute of Functional Neurosurgery, Department of Neurosurgery, Xuanwu Hospital Capital Medical University, Beijing 100053, China; Department of Neurology, Xuanwu Hospital Capital Medical University, Beijing 100053, China; Beijing Institute of Functional Neurosurgery, Department of Neurosurgery, Xuanwu Hospital Capital Medical University, Beijing 100053, China

**Keywords:** neuromodulation, drug-resistant epilepsy, responsive neurostimulation, network modulation

## Abstract

This study aims to investigate the neuromodulatory effects of interictal short-term high-frequency stimulation on both the epileptogenic zone (EZ) and distributed networks, thereby advancing our understanding of the mechanisms underlying the therapeutic efficacy of chronic subthreshold cortical stimulation in refractory epilepsy. Eight patients with epilepsy undergoing stereoelectroencephalography monitoring during pre-surgical evaluation, with periodic spikes observed during the interictal period, were included. Short-term high-frequency stimulation (STHFS) and incremental current stimulation protocols were applied to the EZ. Spike rate, amplitude and spectral power were quantitatively assessed during the interictal (baseline), stimulation and post-stimulation periods. Functional network connectivity was evaluated using the directed transfer function and partial directed coherence. During STHFS, the spike rate, amplitude and spectral power decreased significantly compared with the baseline period. In the post-stimulation period, the spike rate, amplitude and spectral power rapidly rebounded and fluctuated around the baseline. Incremental current stimulation resulted in a gradual reduction in spike rate and spectral power, with maximal inhibition achieved at a specific current intensity. Functional connectivity exhibited individualized patterns across frequency bands during STHFS. In individual patients, specific brain regions consistently demonstrated reduced functional connectivity throughout the stimulation process. Short-term high-frequency stimulation effectively inhibits neuronal excitability in the EZ, with the degree of inhibition dependent on stimulation intensity. STHFS has an anti-epileptic effect in the local EZ region and stably regulates the distributed network.

## Teaser

Yan *et al*. reported that short-term high-frequency electrical stimulation of the epileptogenic zone during the interictal period consistently suppressed spike rate, amplitude and spectral power. Additionally, the regulatory effect of this stimulation extends beyond the local epileptogenic zone and modulates distributed brain networks in individual patients.

## Graphical Abstract

**Graphical Abstract:**
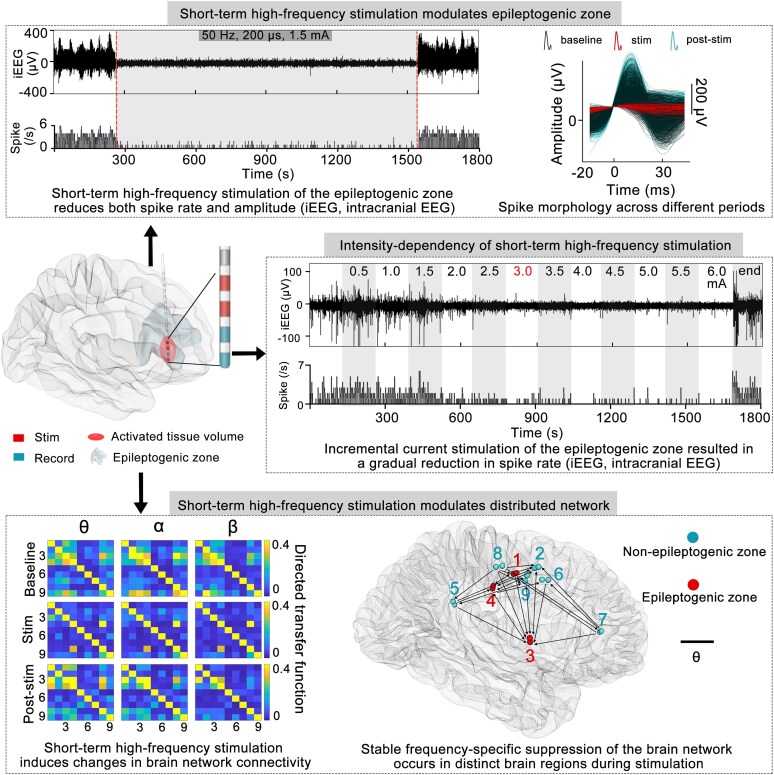

## Introduction

Epilepsy, a common chronic neurological disorder, has a global prevalence of around 1%.^1^ Despite antiseizure medication treatment, ∼30% of patients still experience recurrent seizures, classifying them as having drug-resistant epilepsy.^2^ Although resective surgery is effective for drug-resistant epilepsy, it is limited when the focus is in or near the functional areas. In such cases, resective surgery may lead to neurological deficits (e.g. motor, language or memory impairments), making it a suboptimal treatment option.^3^ For these patients, invasive neuromodulation techniques provide alternative treatment options, including vagus nerve stimulation, deep brain stimulation and responsive neurostimulation (RNS).^4^ Among these, RNS delivers immediate, brief electrical stimulation to the epileptic focus upon detection of epileptiform discharges, aiming to terminate nascent seizure events.^5^ However, Kokkinos *et al*. reported that the indirect effect of RNS was more influential than the direct effect in improving seizure control. They suggested that electrical field barriers created by the interictal cortical electrical stimulation play a key role in preventing the synchronized spread of discharges from the epileptic focus, thereby reducing the occurrence of seizures.^6^ This finding once again raises a controversial question: Can the direct interictal electrical stimulation of the epileptic focus control seizures? While various studies have been conducted to address this question, no definitive conclusions have been reached.^7-13^

Recently, a novel neuromodulation technique known as chronic subthreshold cortical electrical stimulation (CSCS) has been explored to investigate this issue further.^14^ CSCS directly and continuously applies electrical stimulation to the epileptogenic zone (EZ) at an appropriate current intensity during the interictal period to reduce the likelihood of seizures.^15^ Preliminary results from small-sample clinical studies have shown that CSCS is both safe and effective^9,16-21^; however, current clinical research still has not yet clarified the exact mechanism by which CSCS treats epilepsy, limiting its broader clinical application and the optimization of stimulation parameters.

This study aimed to investigate the electrophysiological changes during interictal short-term high-frequency stimulation in patients with epilepsy who have undergone stereoelectroencephalogram (SEEG) electrode implantation. By analysing the differences in electrophysiological signals during the interictal (baseline), stimulation and post-stimulation periods, the study provides insights into the mechanism of CSCS in treating refractory epilepsy. The ultimate goal is to advance the clinical application of CSCS and provide additional interictal stimuli for RNS to form a new multimodal regulatory treatment for epilepsy.

## Materials and methods

### Subjects

The study included patients with epilepsy who sought consultation at the Beijing Institute of Functional Neurosurgery, Xuanwu Hospital, from January 2021 to July 2023. Eleven participants were included based on the following criteria: (i) a confirmed diagnosis of drug-resistant epilepsy^22^; (ii) the requirement for intracranial recordings due to inadequate localizing information; (iii) demonstration of frequent or periodic spikes/slow-spike waves in the suspected EZs during interictal SEEG recordings; and (iv) can effectively communicate and cooperate with the experimental paradigm. Among the participants, two were excluded due to recurrent seizures induced by low-level stimulation (<0.5 mA) of the seizure onset zone (SOZ), while one participant was excluded due to the non-availability of written informed consent.

### Implantation and reconstruction of the SEEG electrode

All patients underwent a comprehensive evaluation by a specialized committee using non-invasive studies. Due to insufficient information on the localization of EZs, resective surgery or radiofrequency thermocoagulation (RFTC) required guidance through SEEG. Experienced neurologists and neurosurgeons designed the number and the trajectory of the SEEG electrodes. The SEEG electrodes (8–20 contacts, length 2 mm, diameter 0.8 mm, spaced 1.5 mm apart) were implanted using the stereotactic method, aided by a robotic arm-assisted system (Sinovation Medical Technology, Beijing, China). The reconstruction and localization of the depth electrodes followed established protocols.^23^

### SEEG recordings and cortical mapping

Long-term SEEG recording was routinely performed with the 128-channel video electroencephalogram (EEG) monitoring system (Micromed, Treviso, Italy) sampled at 1024 Hz. The recording usually lasted 3–14 days to capture at least two habitual clinical seizures. To improve spatial resolution and reduce the influence of widespread signals due to volume conduction, the SEEG data were re-referenced using a bipolar montage.

Electrical cortical stimulation (SD LTM STIM; Micromed) was applied using a bipolar and biphasic pulse configuration on two serial adjacent contacts. This approach mapped the eloquent cortex and helped to locate the SOZ. During the patient's relaxed state in bed, a stimulating current (50 Hz frequency, 0.2 ms pulse width, 3 s duration, current range: 0.1–6 mA) was applied to each pair of contacts. Upon obtaining any response, physicians documented the current threshold, encompassing clinical symptom manifestations (seizure, aura, motor and sensory symptoms) and after discharge. From a safety perspective, we set the maximum stimulation current at 6 mA. If applying 6 mA of electrical stimulation to a specific patient caused no symptoms, this value was considered the threshold current for that patient. Of note, it is essential to validate the threshold currents by repetitively stimulating the contacts (2–3 times).

### High-frequency stimulation protocols

We conducted short-term high-frequency stimulation (STHFS) and incremental current stimulation protocols based on the recorded threshold currents from the mapping procedure (50 Hz frequency, 0.2 ms pulse width, current range: 0.1–6 mA). We selected a pair of contacts situated in the EZ within the grey matter as the stimulating contacts. In addition, these contacts captured the occurrence of periodic spikes or slow-spike waves.

In the STHFS protocol, the stimulating current was adjusted to 90% of the threshold current for patients whose threshold current was <6 mA. Nevertheless, if the determined threshold current for a patient was 6 mA, this value was directly used as the stimulation current. To ensure patient compliance with awake and quiet during stimulation, sessions were maintained for a minimum of 5 min but did not exceed 30 min (adaptable to individual compliance levels). Triplicate stimulation repetitions were performed to reduce intra-individual variability during the protocol. Additionally, it is essential that the patient exhibits no seizure activity for at least 30 min before commencing the stimulation. Throughout the stimulation period, physicians periodically assessed the patients for any abnormalities. After each stimulation period was completed, patients were allocated a resting period of at least 15 min.

In the incremental current stimulation protocol, if the stimulation current for a patient exceeded 2.5 mA (e.g. 6 mA), a fixed step size of 0.5 mA was utilized, allowing for incremental adjustments ranging from 0.5 to 6 mA in 0.5 mA increments. Each current level was administered continuously for a duration of 1–2 min without interruptions. In cases where the stimulation current was <2.5 mA, a fixed step size of 0.2 mA was utilized for stimulation.

### Montage selection and data processing

In the stimulation period, the disconnection of bipolar contacts makes them unsuitable for analysis. Consequently, neighbouring contacts with periodic spikes or slow-spike waves would be selected as recording contacts to represent the EZ. A 5-min block within and after each STHFS period was selected to calculate the spike rate and amplitude. Similarly, three 5-min blocks of interictal SEEG data, obtained from the recording contacts while the patient was awake and quiet, were selected as the baseline measurements. In the dataset concerning incremental current stimulation, differences in stimulation duration arose due to discrepancies in the applied stimulation current among patients. Consequently, we included the entire incremental current stimulation period for all individuals.

The spike count and amplitude measurements were automatically recorded^24^ and subsequently verified by two experienced electrophysiology technicians. The spike rate was determined by calculating the number of spikes recorded per minute. Given that all enrolled patients exhibited inherent periodic spike activity, we retained spikes within the selected epochs for power spectra analysis without performing spike removal. To examine the impact on power across different frequency bands during electrical stimulation, we compared the logarithmically transformed power of the 4–8 Hz (θ), 8–13 Hz (α) and 13–40 Hz (β) bands across various periods. To analyse the trends of power variations at different frequencies across different periods, we investigated the continuous power changes over 5 min (divided into 60 blocks of 5 s each). In the incremental current stimulation dataset, we also investigated the continuous changes in power across different frequency bands as the current gradually increased.

We selected electrode contacts located in the cortex to represent the positions of different brain regions and calculated the directed transfer function (DTF) and partial directed coherence (PDC) to assess the functional network connectivity among these regions during different periods. Both DTF and PDC are rooted in the multivariate time-series analysis framework derived from Granger causality principles, aiming to study directional information flow in neural networks.^25-27^ DTF quantifies the total information flow from one node to others in the frequency domain, encompassing both direct and indirect pathways.^25^ In contrast, PDC evaluates direct effective connectivity between pairwise brain regions by conditioning out the contributions of other network nodes.^28,29^ The two methods were used to investigate the propagation direction and connection strength within epileptic networks.^30,31^ In this study, DTF and PDC were primarily adopted to evaluate the network connection strength across different brain regions during various periods (baseline, stimulation and post-stimulation periods). Values of the DTF and PDC are between 0.0 and 1.0.

A 1-min block within and after each STHFS period was selected to calculate the functional connectivity. Similarly, three 1-min blocks of interictal SEEG data were selected as the baseline measurements. To investigate the effect of STHFS on functional connectivity between the EZ (presence of spikes in the contacts) with non-EZ (NEZ, absence of spikes in the contacts), we performed a comparative analysis of DTF within the EZ (EZ-EZ), within the NEZ (NEZ-NEZ) and between the EZ and NEZ (EZ-NEZ) in individual patients. Additionally, leveraging the advantages of PDC, we calculated the PDC between each pair of contacts to eliminate interference from other brain regions. Then, we compared the PDC of all contacts during the stimulation period (across all three stimulation processes) with the mean PDC in the baseline period to identify network relationships that showed a stable change in the θ, α and β bands.

### Statistics

Data were presented as mean ± standard deviation. Normality was assessed using the Shapiro–Wilk test (normality assumed if *P* ≥ 0.05). Repeated measures ANOVA (RM-ANOVA) followed by Tukey’s multiple comparisons test was performed on data from eight patients to compare spike rates, spike amplitudes and power across the three experimental periods: baseline, stimulation and post-stimulation. For DTF and PDC comparisons across these three periods, parametric data (Shapiro test, *P* ≥ 0.05) were analysed with RM-ANOVA followed by Tukey’s multiple comparisons test, whereas non-normally distributed datasets (Shapiro test, *P* < 0.05) underwent Friedman tests followed by Dunn's multiple comparison test. All statistical analyses were conducted using GraphPad Prism 9.0 (La Jolla, CA, USA), with statistical significance threshold set at *P* < 0.05.

### Ethical statement

All patients provided informed consent after admission (for patients <18 years of age, informed consent was obtained from their parents). The Medical Ethics Committee of Xuanwu Hospital, Capital Medical University, approved this study (LYS2020274).

## Results

### Clinical characteristics of the patients

This study enrolled eight patients, seven males and one female, aged 20.4 ± 6.5 years, who had a history of epilepsy for 12.6 ± 6.3 years. Two of the eight patients presented with motor seizures, and the remaining six experienced focal to bilateral tonic-clonic seizures. Preoperative evaluation localized the EZ to the frontal area in five patients, the parietal area in one patient and the cingulate cortex in two patients. Finally, six of the eight patients underwent resective surgery, while two patients opted for SEEG-RFTC (Table 1).

**Table 1:** Patient summary data

Patient number	Age (years)/sex	Epilepsy duration (years)	Seizure frequency (day)	Epilepsy type	MR findings	Surgery	Pathological diagnosis	Seizure outcome (ILAE classification)
1	23/M	15	3	FBTCS	R-P heterotopia	R-inferior P (SEEG-RFTC)	N	II
2	26/M	5	1/week	FBTCS	R-F signal abnormality	R-inferior F	FCDIIb	I
3	28/Fe	20	1	FBTCS	Negative	R-middle and inferior F	MCD	I
4	17/M	11	3	MS	R-F signal abnormality	R-middle and inferior F	FCDIIb	I
5	7/M	2	5	MS	R-middle F cortical thickening	R-middle and superior F	FCDI	I
6	22/M	17	3	FBTCS	Negative	L-inferior F and medial F	FCDIIa	I
7	18/M	14	1	FBTCS	Negative	R-MCC (SEEG-RFTC)	N	II
8	22/M	17	2/month	FBTCS	Negative	R-superior F and ACC	MCD	I

FBTCS, focal to bilateral tonic-clonic seizures; FCD, focal cortical dysplasia; MCD, malformative cortical dysplasia; MS, motor seizure; SEEG, stereoelectroencephalogram; RFTC, radiofrequency thermocoagulation; F, frontal; ACC, anterior cingulate cortex; MCC, middle cingulate cortex; P, parietal; L, left; R, right; M, male; Fe, female.

The number of implanted electrodes per patient was 6.9 ± 1.8 (range: 4–9), with a total number of 91.0 ± 25.2 (range: 52–124) contacts. All stimulation and recording contacts were located within the EZ (surgical resection or RFTC area, Supplementary Fig. 1). Four of the eight patients had both the stimulation current and the threshold current set at 6 mA. For the remaining four patients, the stimulation current (2.55 ± 1.35 mA) was set to 90% of the threshold current (2.85 ± 1.50 mA; Table 2).

**Table 2:** Patient electrode distribution and current settings

Patient number	Electrodes/contacts	Distribution of contacts	Contacts in EZ	Stimulating region	Threshold current (mA)/stimulating symptom	Stimulating current (mA)
1	5/90	Record contacts (1), SMA (2), F (3), paracentral lobule (4), P (6, 7, 9, 11), T (8), postcentral gyrus (5,10)	1, 4, 5, 6, 7, 9, 11	R-inferior P	6/–	6
2	9/124	Record contacts (12), hippocampus (1), T (2), ACC (3), F (4, 5, 6, 8, 13), insula (7, 9), P (10), MCC (11), SMA (14), precentral gyrus (15)	9, 12	R-inferior F	1.7/numbness in left arm	1.5
3	4/62	F		R-superior F	6/–	6
4	6/52	F		R-middle F	5/numbness in left hands	4.5
5	7/114	Record contacts (7), MCC (1), precentral gyrus (2), SMA (3), F (4, 6, 9), ACC (5), insula (8), hippocampus (10), T (11)	2, 3, 4, 7	R-superior F	6/–	6
6	8/98	Record contacts (2), F (1, 3, 5), ACC (4), insula (6), precentral gyrus (7), hippocampus (8), T (9)	1, 2, 3	L-medial F	6/–	6
7	9/80	Record contacts (4), MCC (1), F (2, 6, 9), Insula (3), PCC (5), ACC (7), SMA (8)	1, 3, 4	MCC	2/numbness in left arm	1.8
8	7/108	Record contacts (4), F (1, 2, 5, 7, 12), ACC (3), insula (6), hippocampus (8), T (9, 10, 11)	4, 5, 7	ACC	2.6/a sense of nervousness	2.4

ACC, anterior cingulate cortex; F, frontal; MCC, middle cingulate cortex; PCC, posterior cingulate cortex; P, parietal; T, temporal; SMA, supplementary motor area; L, left; R, right; EZ, epileptogenic zone.

### Spike rate and amplitude changes

**Figure 1:**
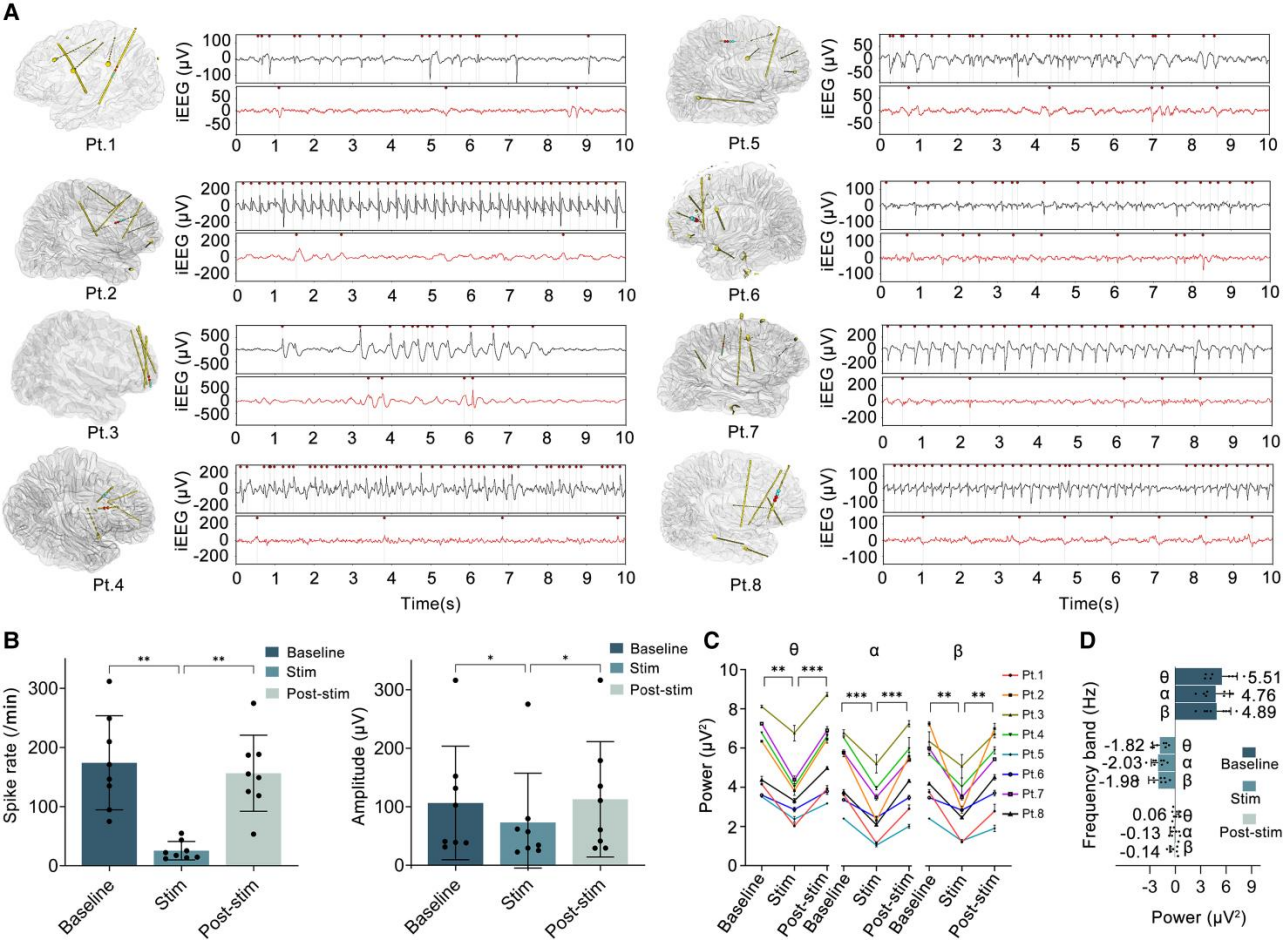
**Changes in spike rate, amplitude and power across different periods in the eight patients.** (**A**) The left image shows the positional relationship in the brain between stimulation contacts (red dots) and recording contacts (blue dots) for the eight patients. The right upper image displays periodic spikes or slow-spike waves (red point) recorded during a 10-s baseline block. The right lower image shows a significant reduction in both the number and amplitude of spike during the 10-s stimulation block. (**B**) Changes in spike rate and amplitude in patients during the baseline, stimulation and post-stimulation period. Both parameters were analysed with RM-ANOVA followed by Tukey’s multiple comparisons test (*n* = 8, spike rate: *F* = 24.88, *P* = 0.0008, *P*_1_ = 0.0040, *P*_2_ = 0.3180, *P*_3_ = 0.0023; spike amplitude: *F* = 8.331, *P* = 0.0125, *P*_1_ = 0.0281, *P*_2_ = 0.6700, *P*_3_ = 0.0487; *P*_1_, *P*_2_, *P*_3_ represent baseline versus stimulation, baseline versus post-stimulation, stimulation versus post-stimulation, respectively). Data points represent the mean spike rate or amplitude during specific experimental periods (baseline, stimulation and post-stimulation) for each patient. (**C**) Power changes in different frequency bands in the patients during the baseline, stimulation and post-stimulation period using RM-ANOVA followed by Tukey’s multiple comparisons test (*n* = 8, θ band: *F* = 39.89, *P* < 0.0001, *P*_1_ = 0.0012, *P*_2_ = 0.9125, *P*_3_ = 0.0004; α band: *F* = 44.78, *P* < 0.0001, *P*_1_ = 0.0005, *P*_2_ = 0.7174, *P*_3_ = 0.0004; β band: *F* = 21.54, *P* = 0.0008, *P*_1_ = 0.0048, *P*_2_ = 0.7106, *P*_3_ = 0.0042; *P*_1_, *P*_2_, *P*_3_ represent baseline versus stimulation, baseline versus post-stimulation, stimulation versus post-stimulation, respectively). Data points represent the mean power within specific frequency bands during experimental periods (baseline, stimulation and post-stimulation) for each patient. (**D**) The upper graph shows the mean power in different frequency bands during the baseline period. The middle and lower graphs show the changes in power across all frequency bands relative to baseline during the stimulation and post-stimulation periods, respectively (RM-ANOVA, *n* = 8, stimulation period: *F* = 0.1117, *P* > 0.05; post-stimulation period: *F* = 0.4846, *P* > 0.05). Data points represent the mean power/the extent of power decrease in specific frequency bands in each patient. Pt., patient; iEEG, intracranial EEG. *P < 0.05, **P < 0.01, ***P < 0.001, ****P < 0.0001.

**Figure 2:**
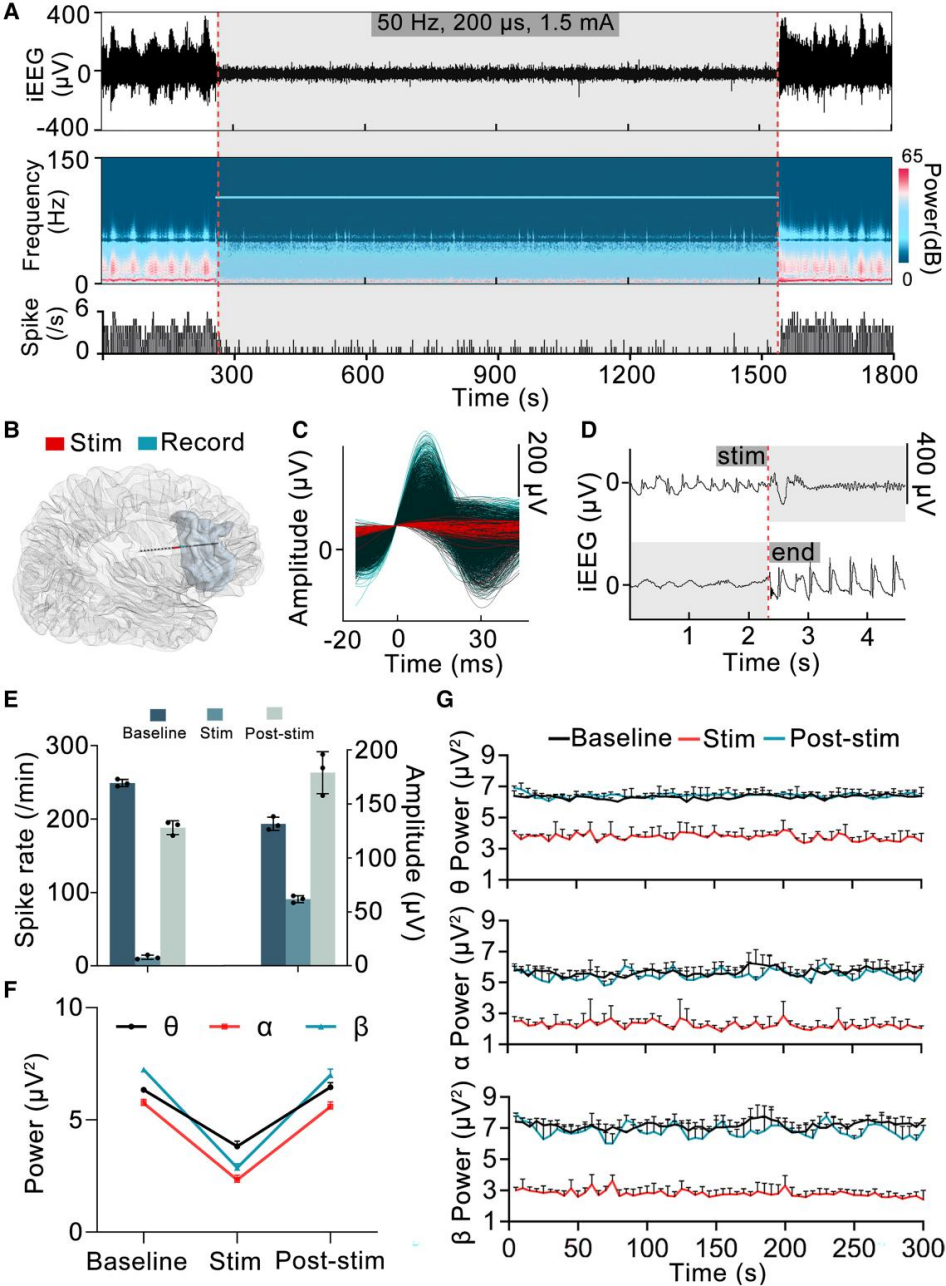
**Changes in spike waves and power for Patient 2 across different periods.** (**A**) The upper image shows the iEEG of Patient 2 during the pre-stimulation, stimulation and post-stimulation periods. The middle graph shows the corresponding time-frequency representation. The lower graph displays the spike numbers per second. (**B**) The spatial relationship between the EZ (grey) and the stimulation and recording contacts of Patient 2. (**C**) Changes in the morphology of spikes during the baseline (black), stimulation (red) and post-stimulation (blue) periods. (**D)** Instantaneous inhibition and recovery of spikes can be observed at the onset and offset of stimulation. (**E)** The spike rate and amplitude in Patient 2 across the different periods. Data points represent the mean spike rate or amplitude in each five-min block. (**F)** Changes in the power of θ, α and β bands in Patient 2 across the different periods. Data points represent the mean power within specific frequency bands during experimental periods (baseline, stimulation and post-stimulation). **(G)** The power of the θ, α and β bands showed a stable decrease during the 5-min stimulation period, while the power of each frequency band fluctuated around baseline during the post-stimulation period. Data points reflect 5-s averaged power values in specific frequency band extracted from predefined 5-min blocks (baseline/stimulation/post-stimulation). iEEG, intracranial EEG; EZ, epileptogenic zone.

Periodic spikes were observed at recording contacts during the baseline period in all enrolled patients (Fig. 1A, Supplementary Fig. 2). Seven of the eight patients received STHFS procedures three times, while one patient received two (Patient 4). The mean spike rate of the patients during the baseline period is 174.11 ± 79.59/min. In contrast to the baseline period, the spike rate during stimulation decreased significantly in the eight patients (Fig. 1A and B), averaging 25.43 ± 15.61/min (*P* < 0.01), with a mean reduction of 82.49% (range: 62.52–95.61%; Fig. 1B, Supplementary Table 1). During the stimulation period, the spike amplitude significantly differed from the baseline period (73.17 ± 84.14 versus 106.44 ± 96.99 μV, *P* < 0.05), reflecting a mean decrease of 32.16% (range: 10.22–52.99%; Fig. 1B). The spike rate and amplitude were stably inhibited during the stimulation period (Fig. 2A). Within 5 min of the post-stimulation period, the spike rate and amplitude were both elevated in comparison with those during the stimulation period (*P* < 0.01, *P* < 0.05). In contrast to the baseline period, the spike rate and amplitudes during the post-stimulation period exhibited varying trends among the patients, although not significant (both *P* > 0.05; Fig. 1B). Additionally, at the start of the stimulation procedure, we observed an immediate inhibition of both the spike rate and amplitude in all patients; however, upon cessation of stimulation, both the spike rate and amplitude showed immediate recovery (Fig. 2A and D).

### Spectral power changes

In contrast to the baseline period, significant reductions in spectral power were observed across the frequency bands of 4–8 Hz (*P* < 0.01), 8–13 Hz (*P* < 0.01) and 13–40 Hz (*P* < 0.01) during the stimulation period (Fig. 1C). However, there was no statistically significant difference in the degree of power decrease across the θ (33.12%), α (44.64%) and β (40.69%) bands during stimulation (*P* > 0.05; Fig. 1D). The spectral power in different frequency bands during the post-stimulation period was significantly elevated compared with the stimulation period (*P* < 0.01) but showed no statistical differences compared with the baseline period (*P* > 0.05). During the 5-min stimulation period, the power in the θ, α and β bands stably decreased in all eight patients. After the stimulation ceased, the power in the different frequency bands increased rapidly and fluctuated around baseline (Fig. 2G, Supplementary Fig. 3).

### Spike and power changes during incremental current stimulation

**Figure 3:**
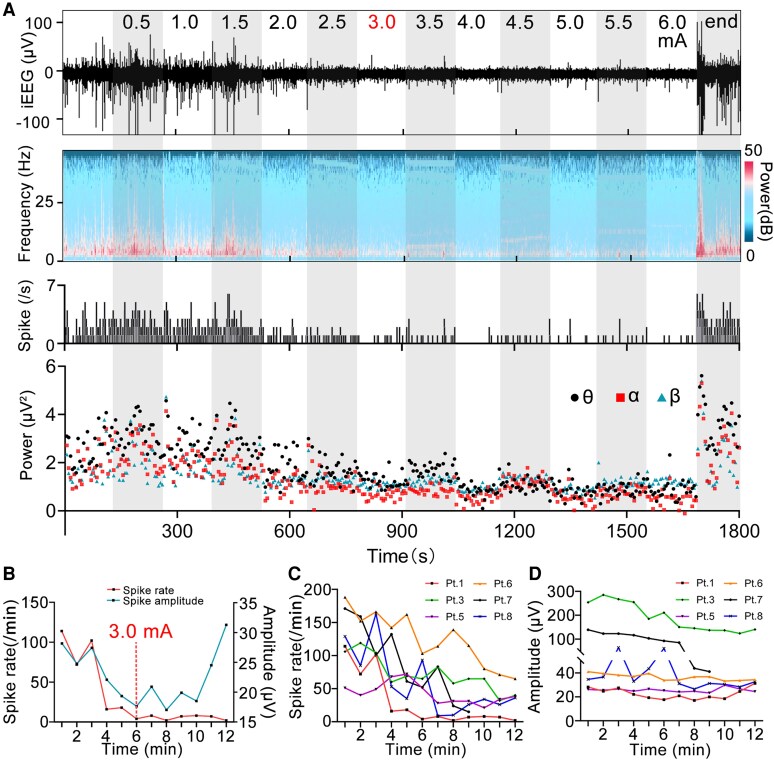
**Changes in spike and power during incremental current stimulation.** (**A**) The upper image shows that the number and amplitude of spikes in Patient 1 were gradually inhibited during incremental current stimulation and remained stable after stimulation at 3.0 mA. The middle graph shows the corresponding time–frequency diagram and the number of spikes per second. The lower graph shows the power in different frequency bands during the incremental current stimulation was decreased gradually and was stable after the stimulation at 3.0 mA. Data points represent 5-s averaged power values within specific frequency. (**B**) Changes in the spike rate and amplitude of Patient 1 during the stimulation process (1 min block was selected at each stimulation intensity). (**C** and **D**) The changes of the spike number and amplitude of six patients during the incremental current stimulation, respectively. In **B–D** data points represent the average spike rate or amplitude in each one-min block. iEEG, intracranial EEG.

**Figure 4:**
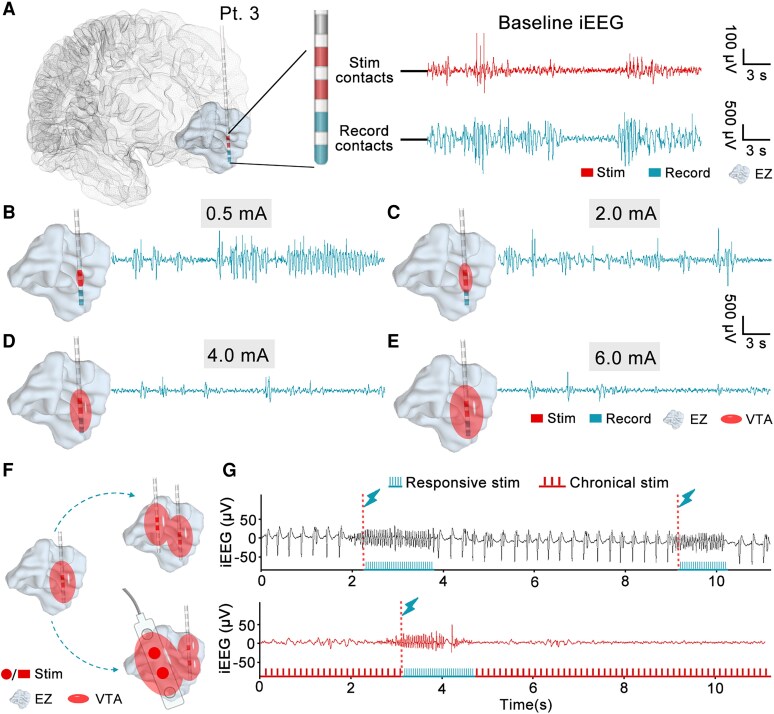
**The impact of current-induced changes in VTA on spike activity within the EZ.** (**A**) In Patient 3, the EZ is located in the middle and inferior frontal gyrus. The electrode is positioned within the EZ, with stimulation and recording contacts adjacent to each other, and frequent spikes are recorded during the baseline period. (B–E) Simulated current-dependent VTA changes and their impact on spike activity in the EZ. (**B)** At 0.5 mA, the VTA is confined to the stimulation contacts, with no inhibitory effect on the EZ near the recording contacts. (**C**) At 2 mA, the VTA partially covers the EZ around the recording contacts, leading to a reduction in both the number and amplitude of spikes. (**D)** At 4.0 mA, the VTA almost fully covers the EZ near the recording contacts, resulting in a further inhibition of spikes. (**E**) At 6.0 mA, although the VTA increased furtherly, no additional inhibitory effect occurs. (**F**) The left image shows that the VTA generated by a single depth electrode is insufficient to cover the entire EZ. Therefore, multiple depth electrodes (right-upper) or a combination of strip electrodes and depth electrodes (right-lower) can be used to improve the volume ratio between the VTA and EZ. (**G**) The upper image shows the stimulation paradigm of RNS. The lower image depicts the multimodal neuromodulation approach, combining responsive stimulation and a chronical stimulation pattern. Pt., patient; iEEG, intracranial EEG; EZ, epileptogenic zone; VTA, activated tissue volume.

Six of the eight patients received the incremental current stimulation protocol (patient 1, 3, 5, 6, 7 and 8). As the stimulation intensity increased, we observed a gradual reduction in both the spike rate and power across different frequency bands (Figs 3A–C and 4B–E). Interestingly, according to the visual analysis, all patients achieved maximum suppression of spike rate and power at a lower current than the peak stimulation intensity (Fig. 3A and B, Supplementary Fig. 4). Despite the increase in current and corresponding decrease in spike rate, some patients did not exhibit the expected reduction in spike amplitude due to its inherent instability (Fig. 3B and D).

### Changes in the distributed network

**Figure 5:**
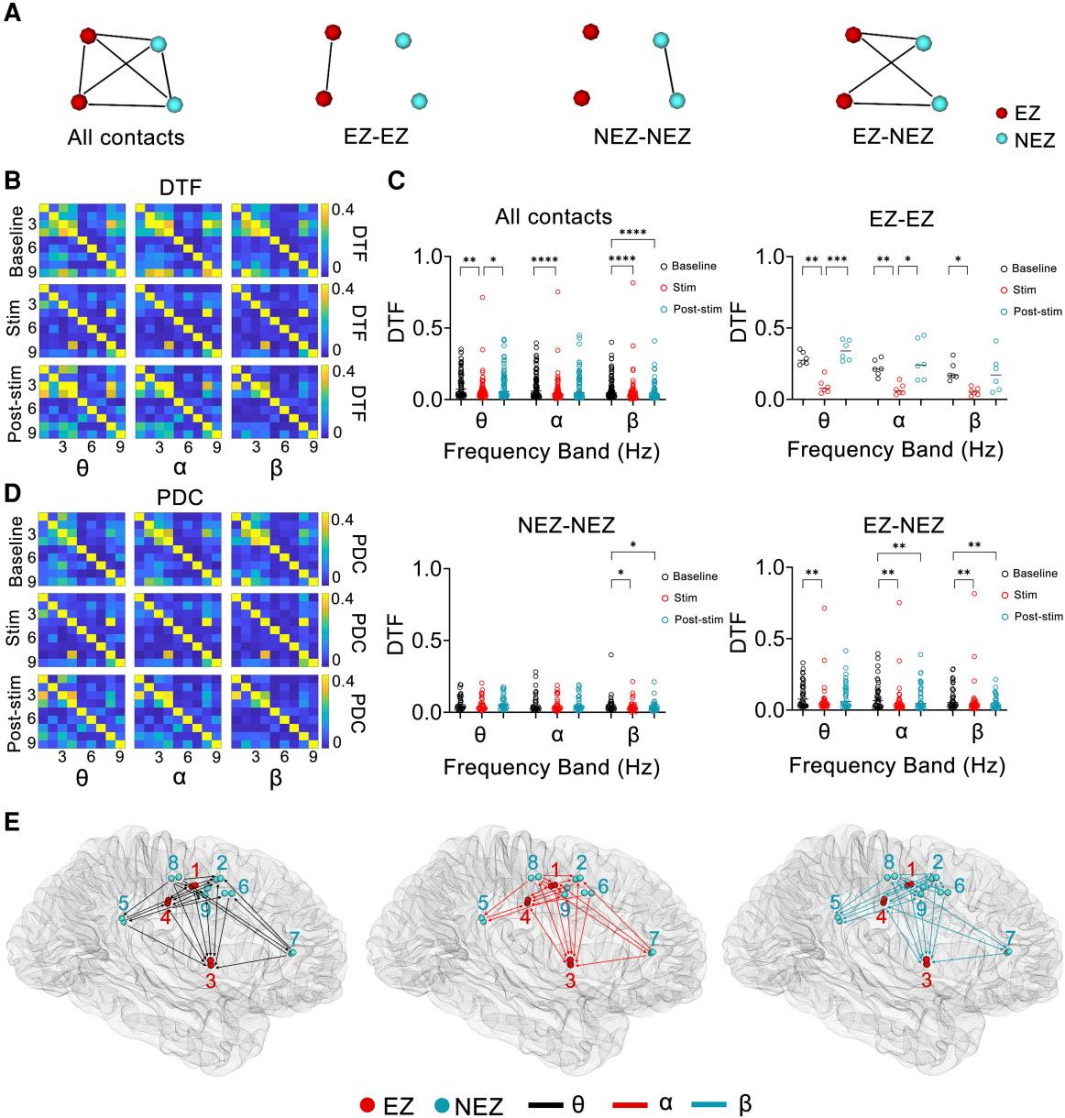
**The effect of short-term high-frequency stimulation on the brain network in Patient 7.** (**A**) Schematic diagram of network connections in different groupings. (**B** and **D**) Heat maps of DTF and PDC values across different periods (baseline/stimulation/post-stimulation) and frequency bands; in **B** and **D**, the numerical labels on the *x*- and *y*-axes of each matrix represent the electrode contacts within selected brain regions (number of contacts = 9). Each data point indicates the DTF or PDC value from the source (*y*-contact) to the target (*x*-contact) (*n* = 81). (**C**) Comparisons of DTF between baseline, stimulation and post-stimulation periods within specific frequency bands (θ, α and β) and connection groupings (all contacts, EZ-EZ, NEZ-NEZ, EZ-NEZ). Parametric data (Shapiro test, *P* ≥ 0.05) were analysed with RM-ANOVA followed by Tukey’s multiple comparisons test, whereas non-normally distributed datasets (Shapiro test, *P* < 0.05) underwent Friedman tests followed by Dunn's multiple comparison test (all contacts, *n* = 72, θ band: $\chi_{F}^{2}$ = 14.53, *P* = 0.0007; α band: $\chi_{F}^{2}$ = 17.36, *P* = 0.0002; β band: $\chi_{F}^{2}$ = 28.58, *P* < 0.0001. EZ-EZ, *n* = 6, θ band: *F* = 31.84, *P* = 0.0005; α band: *F* = 13.95, *P* = 0.0040; β band: *F* = 5.494, *P* = 0.0346; NEZ-NEZ, *n* = 30, θ band: $\chi_{F}^{2}$ = 1.667, *P* = 0.4346; α band: $\chi_{F}^{2}$ = 3.267, *P* = 0.1953; β band: $\chi_{F}^{2}$ = 9.867, *P* = 0.0072. EZ-NEZ, *n* = 36, θ band: $\chi_{F}^{2}$ = 12.50, *P* = 0.0019; α band: $\chi_{F}^{2}$ = 15.17, *P* = 0.0005; β band: $\chi_{F}^{2}$ = 15.06, *P* = 0.0005). *P < 0.05, **P < 0.01, ***P < 0.001, ****P < 0.0001. Each data point corresponds to the mean DTF value calculated between paired contacts within defined frequency bands and temporal intervals. (**E**) Contact pairs in which PDC during the three stimulation processes across different frequency bands were lower than the average PDC during the baseline period. Red and blue contacts in the brain model denote the EZ and NEZ, respectively. Solid lines between contacts indicate connectivity, with arrowheads specifying the direction of PDC (from source to target contact). EZ, epileptogenic zone; NEZ, non-epileptogenic zone; DTF, directed transfer function; PDC, partial directed coherence.

According to the electrode distribution, the electrodes in two patients were confined to EZ (Patients 3 and 4). Therefore, we calculated the functional connectivity between EZ and NEZ of the remaining six patients. On average, 8.5 ± 3.6 pairs of contacts representing different brain regions were selected for each patient. Compared with the baseline and post-stimulation periods, visual analysis of DTF and PDC heat maps during the stimulation period suggested a decreasing trend in network connectivity within specific frequency bands in individual patients (Fig. 5B and D, Supplementary Figs 5 and 6). However, statistical analysis revealed significant differences in only a small subset of patients (Fig. 5C, Supplementary Figs 5 and 6). For each patient, we calculated DTF changes (ΔDTF = stimulation DTF − baseline DTF) across all contacts in different frequency bands and determined the median of these changes. Negative median values indicate a decreasing trend in connectivity, while positive values indicate an increasing trend. The results revealed decreasing connectivity trends in four patients (θ band), three patients (α band) and two patients (β band; Supplementary Table 2). However, no consistent DTF changes in specific frequency bands were found across all patients.

The average number of contact pairs in the EZ and NEZ were 3.6 ± 1.8 and 7.5 ± 3.1, respectively. Based on the DTF changes analysis, the decreasing trends of network connectivity across different frequency bands during stimulation exhibited the following distribution among different contact pairs of the patient: EZ-EZ pairs showed decreasing trends in three patients (θ), three patients (α) and four patients (β); NEZ-NEZ pairs in three patients (θ), three patients (α) and three patients (β); and EZ-NEZ pairs in four patients (θ), five patients (α) and four patients (β) (Supplementary Table 2). Similar to changes in the overall brain network (all contacts), the DTF among different patients in the EZ-EZ, NEZ-NEZ and EZ-NEZ contact pairs across different frequency bands exhibited individualized patterns (Fig. 5C, Supplementary Figs 5 and 6).

We analysed the contact pairs in six patients whose PDC during the three stimulation periods were consistently lower than their average PDC during the baseline period. A total of 32.5 ± 8.9 contact pairs showed stable decreases in the θ band, 33.0 ± 10.4 in the α band and 36.8 ± 10.8 in the β band (Fig. 5E, Supplementary Fig. 7). In these contact pairs, no significant difference was observed between the PDC in the post-stimulation and baseline periods (*P* > 0.05). No uniform PDC changes in specific frequency bands or brain regions were observed across all patients.

## Discussion

In this study, we report that STHFS of the EZ during the interictal consistently suppressed the spike rate and amplitude, and significantly inhibited spectral power. Additionally, the inhibitory effect of cortical electrical stimulation was found to be intensity dependent. Furthermore, the regulatory effect of STHFS is not limited to the local EZ region and can modulate the distributed brain network of individual patients.

### Electrical stimulation inhibits the spike rate and amplitude

In this study, the spikes exhibited periodicity during the interictal period; however, when the STHFS was performed on the EZ, the spike rates and amplitude decreased. Interictal spikes result from synchronized paroxysmal depolarization in highly excitable neuron assemblies.^32,33^ Cortical electrical stimulation exerts a direct and sustained inhibitory effect on these assemblies within the EZ, preventing the excitatory neuron assemblies from reaching high levels of synchronization.^34^ The rapid rebound of spike numbers and amplitude following stimulation cessation further supports the significant inhibitory effect of electrical stimulation. Of note, the inhibitory effect on spikes occurred rapidly at the start of stimulation, which may be related to high-frequency stimulation quickly inactivating voltage-gated sodium channels, thereby reducing neuronal excitability.^34,35^

### Electrical stimulation inhibits spectral power

Due to the periodic spike characteristics of the enrolled patients, it was challenging to exclude spike components when calculating the spectral power across different frequency bands. Although we observed a significant and continuous inhibitory effect on spectral power during STHFS, accompanied by a marked reduction in spike rate, this phenomenon may confound the comparison of background power across frequency bands between the baseline and stimulation periods. Specifically, pronounced spike suppression during the stimulation period may mask frequency-specific alterations in background power during spectral decomposition analyses. The study by Westin *et al*. demonstrated a significant decrease in spike rates of SOZ during CSCS.^36^ Furthermore, after removing spike-related components, they calculated the background power and identified significant reductions in θ, α and β band power during stimulation compared with the baseline. In this study, despite differences in stimulation parameters (50 versus 2 Hz) compared with those used by Westin *et al*., we similarly observed a decline in spike rates during stimulation periods, as well as reduced power across different frequency bands (including spike components in our analysis). Based on these findings, we hypothesize that STHFS may simultaneously suppress spike rates within the EZ and inhibit background power in the θ, α and β bands in this region.

Similar to the trends of spike rates and amplitude in the post-stimulation period, the power in each frequency band quickly returned to baseline following the cessation of stimulation. Previous studies reported that cortical stimulation has an inhibitory effect lasting for several minutes, hours, days, even years in the post-stimulation period^7,16,37^; however, in our study, patients received only short-term (5–30 min) stimulation, and a continuous inhibitory effect during the post-stimulation period was not observed. We hypothesize that a longer duration of stimulation may be necessary to induce and sustain a more prolonged inhibitory effect during the post-stimulation period. In contrast to the short-lived suppression of cortical excitability caused by STHFS, long-term stimulation may gradually impair the ability of neuronal assemblies to reach a high level of synchronization.^6^

### Intensity-dependency of the stimulation effect

As the current intensity gradually increased during the stimulation process, we observed a gradual decrease in the spike rate and spectral power. This phenomenon may be related to the gradual expansion of the activated tissue volume (VTA) as the current intensity increases. The VTA is a computational estimate of the neural tissue region where extracellular electrical stimulation induces axonal depolarization, resulting in action potential generation.^38^ In the VTA, the region closer to the stimulation contacts exhibits a stronger current density, leading to the greater inhibition of nearby neurons.^39,40^

In this study, the stimulation and recording contacts were located close to each other within the EZ. Taking Patient 3 as an example (Fig. 4A–E), as the stimulation intensity increased from 0.5 to 4 mA, the VTA enlarged, resulting in a progressively stronger electric field around the recording contacts, which gradually inhibited the excitability of nearby neurons. This inhibition makes it progressively more difficult for frequent, high-amplitude synchronous discharges to form. As the current was gradually increased to a specific value (4 mA), the electric field distribution within the VTA completely covered the recording contacts, fully inhibiting the excitability of the surrounding neuron assemblies. Therefore, further increasing the stimulation intensity (from 4 to 6 mA) was unlikely to produce additional inhibitory effects. Kokkinos *et al*. suggested that electrical stimulation of the EZ can create electrical field barriers that isolate excitatory neuron groups.^6^ Our study demonstrates that the neural excitability of the local region of the EZ is gradually inhibited by the electric field as the current intensity increases, providing additional support for this hypothesis.

### Electrical stimulation regulates the distributed network

Epilepsy is considered a brain network disorder.^41^ Based on the concept of neural connectivity plasticity, the brain network may undergo reorganization during multiple seizures.^42,43^ Similarly, transforming the epileptic brain network into a healthy state through neuromodulation techniques is a chronical process. Recent studies have found that the RNS functions not only in the onset of seizures but also exerts regulatory effects via substantial stimulation in the interictal period.^6,44^ The long-term improvement of seizures in patients with epilepsy may be related to the stimulation carried out in the brain state with less epileptiform activity.^44^ Furthermore, clinical studies of RNS for epilepsy have shown that patients with significant long-term improvements (>90%) exhibit functional network reorganization, characterized by reduced functional connectivity in the α and β bands and enhanced connectivity in the γ bands between their SOZ.^45^ In this study, we conducted short-term high-frequency stimulation on the EZ. Furthermore, due to the differences among patients in the location of the EZ and the distribution of electrodes, we failed to observe the long-term functional connectivity changes consistent with previous studies. However, the individualized changes in network connectivity presented in different periods, frequency bands and brain regions in individual patients, indicating that the regulatory effect of STHFS is not only limited within the local region of the EZ but can also regulate the distributed brain network of individual patients. It is worth noting that during the stimulation period, the connectivity between certain brain regions showed a stable decrease (three stimulation processes) in individual patients. Although it is difficult to determine whether the functional connectivity changes in this study are related to improvements in seizures, the stable connectivity changes induced by short-term stimulation may serve as a prerequisite for network connectivity reorganization driven by long-term interictal stimulation.

Interictal STHFS of the EZ can stably regulate the excitability of neuron assemblies and distributed networks during the stimulation period. Therefore, we hypothesize that long-term cortical stimulation could maintain the local region of the EZ and related brain network in a stable and passive regulatory state. Over time, cortical stimulation could gradually reduce the synchronized neuronal activity in the core of the EZ, disrupt the connectivity of the epileptogenic network, promote the reorganization of the brain's functional network towards a healthier state, and ultimately decrease the severity of seizures.^6,44^

### Enlightenments for clinical application

In previous clinical applications of CSCS, significant variability has been observed in the selection of stimulation parameters across individual patients.^9,14,16,18,46^ Notably, High-frequency stimulation has demonstrated notable efficacy in certain patients with epilepsy.^9,14,16,18,46^ In contrast to the higher stimulation frequencies and longer pulse durations (60–130 Hz, up to 450 µs) employed in clinical practice, this study prioritized safety considerations and thus refrained from testing higher frequencies, and the functional mapping parameters (50 Hz frequency, 0.2 ms pulse duration) was used to the short-term stimulation. Future studies should systematically evaluate whether augmenting stimulation frequency could enhance the spike suppression efficacy in patients with periodic spikes.

Importantly, CSCS with low-frequency stimulation has also shown therapeutic potential in refractory epilepsy.^14,18,46^ Child *et al*. documented that high-frequency stimulation exacerbated epileptiform activity in one patient, whereas low-frequency stimulation (1 Hz, 450 ms pulse width) reduced seizure frequency.^18^ Paradoxically, another patient exhibited seizure burden escalation with low-frequency stimulation but suppression with high-frequency stimulation.^18^ These findings suggest substantial inter- and intra-individual heterogeneity in therapeutic responses to frequency-specific stimulation, which could be attributable to patient- or disease-specific factors and might further correlate with the spatial relationship between the stimulation field and EZ architecture.^18,19^ A limitation of this study is the absence of low-frequency stimulation protocols, precluding direct evaluation of its effects on patients with periodic spikes. Future investigations incorporating comparative low-frequency or higher-frequency stimulation protocols are warranted to elucidate.

In clinical applications of CSCS, the reduction of interictal spikes, a phenomenon considered beneficial,^14,46^ may be associated with short-term therapeutic efficacy.^46,47^ All patients enrolled in this study exhibited periodic spikes, and STHFS led to a significant reduction in spike rate. Although seizure outcomes could not be evaluated due to the study's short-term stimulation protocol, we hypothesize that high-frequency stimulation could provide a potential reference parameter for CSCS in patients with periodic spikes localized within the EZ.

Notably, among the initial cohort of 10 patients investigated, 2 patients (20%) were withdrawn from the study due to recurrent seizures triggered by low-intensity stimulation (<0.5 mA). In the remaining enrolled patients, we observed interindividual variability in threshold currents (range: 1.7–6 mA). This variability may be attributed to the location of the stimulating electrode contacts and the intrinsic properties of each patient's EZ. These factors likely contributed to an exceptionally low threshold current in the two excluded patients. Consequently, even though the stimulation current applied to the EZ was low intensity, it may still have exceeded the individual threshold current, thus potentially triggering seizure recurrence. Furthermore, we cannot exclude the possibility that high-frequency stimulation may not be well tolerated in these two specific patients. These findings underscore the necessity of trial stimulation prior to permanent CSCS implantation in clinical practice. On the other hand, this phenomenon suggests that particular caution is warranted when considering high-frequency stimulation therapy for patients exhibiting periodic spikes.

In clinical trials investigating CSCS, this therapeutic approach has demonstrated significant efficacy in specific types of epilepsy. Previous studies have shown favourable outcomes in cases where the EZ overlapped with functional areas.^17,20^ Freigang *et al*. reported that CSCS (referred to as ‘subacute neocortical stimulation’ in their study) reduced seizure frequency in patients with focal cortical dysplasia.^9^ Similarly, Valentin *et al*. observed marked therapeutic effects following CSCS in patients with epilepsia partialis continua,^16^ and in another study, CSCS applied to paediatric epilepsy populations was shown to be effective and well tolerated.^19^ Prior research has established a preliminary framework for patient selection in CSCS clinical applications based on factors such as EZ localization and pathological types. Although our study lacks seizure outcome data, the observed significant reductions in spike rates during STHFS suggest that periodic spikes may serve as a potential biomarker for patient selection in the clinical application of CSCS.

In this study, STHFS induced stable, individual-specific alterations in brain network. While the limited sample size in this study necessitates further cohort expansion, methodological refinement and external validation, these preliminary findings suggest a potential framework for advancing personalized clinical applications of CSCS. Specifically, monitoring patient-specific brain network alterations through experimental stimulation paradigms may aid in optimizing stimulation parameters and electrode placement for personalized therapeutic regimens. Importantly, this approach necessitates systematic investigation into the modulation effects of STHFS of the EZ on specific brain regions at the individual patient level.

Currently, stimulation intensity is typically determined by clinicians through repeated trials based on the patient's clinical response. The use of biomarkers, such as the spike rate or spectral power, combined with an understanding of the intensity dependency of stimulation effects, can help clinicians optimize settings to create a VTA that adequately covers the EZ while minimizing the risk of side effects from excessive current intensity. For example, employing multiple depth electrodes or combining strip electrodes and adjusting their orientation and placement can help generate a VTA that fully encompasses the EZ (Fig. 4F). However, this approach requires further research to better understand the relationships between stimulation sites, intensity, VTA and the volume of the EZ.

Given the efficacy of CSCS and RNS in treating epilepsy, the question remains whether it is possible to integrate both treatment modalities into a multimodal treatment paradigm. We propose a multimodal neuromodulation approach that could provide chronic stimulation during the interictal period to modulate the excitability of the EZ and the distributed brain network; it could also detect real-time seizure onset and deliver responsive stimulation to prevent impending seizures (Fig. 4G). Although further refinement of the regulation strategies is needed and there are challenges in equipment engineering, we are confident that this multimodal neuromodulation approach can lead to better treatment outcomes.

### Limitations

Our study has certain limitations. First, the limited cohort size may constrain the generalizability of stimulation outcomes. Future studies should expand sample sizes to validate and extend these findings, particularly to examine the modulation effects of high-frequency stimulation of the EZ on specific brain regions at the individual patient level. Second, all patients exhibited periodic or frequent spikes, and future studies should investigate the impact of cortical electrical stimulation on other discharges or epilepsy types. Third, the current investigation focused exclusively on short-term 50 Hz stimulation targeting the EZ. Systematic evaluation of alternative frequency parameters remains necessary, and future protocols should incorporate longer stimulation periods while including seizure frequency as an efficacy metric for stimulation outcomes. Fourth, the volume of the EZ and the relative position of the electrodes within the EZ varied among patients, limiting the ability to assess the degree of VTA-EZ matching in specific patients. Finally, depth electrodes were used to investigate the stimulation effect; however, differences in stimulation parameters and inhibitory effects may exist compared with strip or sheet electrodes.

## Conclusion

In conclusion, interictal STHFS can exert an anti-epileptic effect by inhibiting the excitability of neurons in the EZ with the degree of inhibition depending on the stimulation intensity. In addition, STHFS not only has an anti-epileptic effect in the local region of the EZ but also stably regulates distributed brain networks across different regions.

## Supplementary Material

fcaf378_Supplementary_Data

## Data Availability

The data that support the findings of this study are available on request from the corresponding author. The data are not publicly available due to privacy and ethical restrictions.
