# Supplementary material for: Interictal short-term high-frequency cortical stimulation modulates epileptogenic zone and distributed network
Source: Brain Commun. 2025 Sep 30;7(5):fcaf378. doi: 10.1093/braincomms/fcaf378 (PMC12527290; doi:10.1093/braincomms/fcaf378)
Supplement: fcaf378_Supplementary_Data [file fcaf378_supplementary_data.pdf]

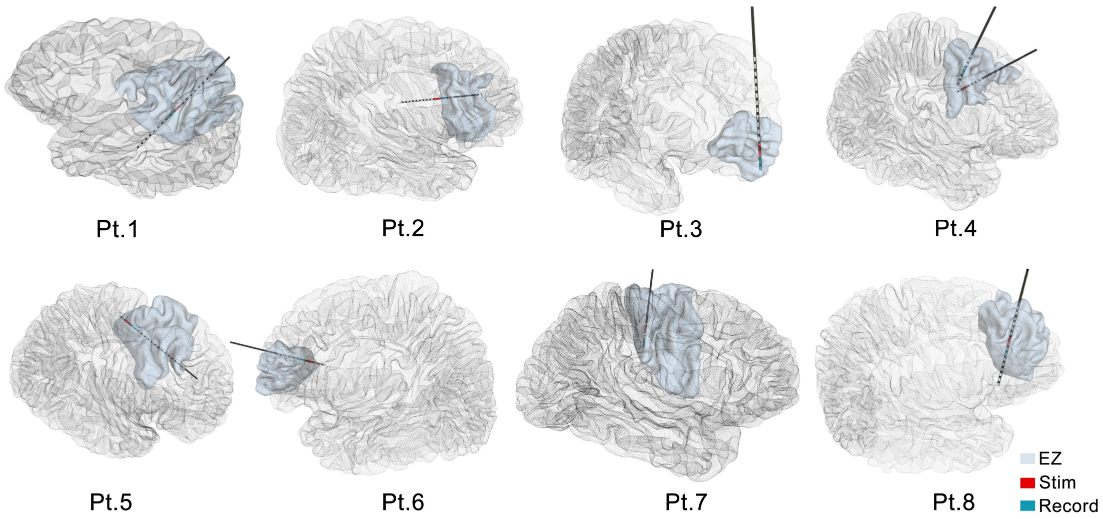

Supplementary Fig. 1 Spatial relationship between the EZ and stimulation/recording electrodes in eight patients. Pt., patient; EZ, epileptogenic zone.

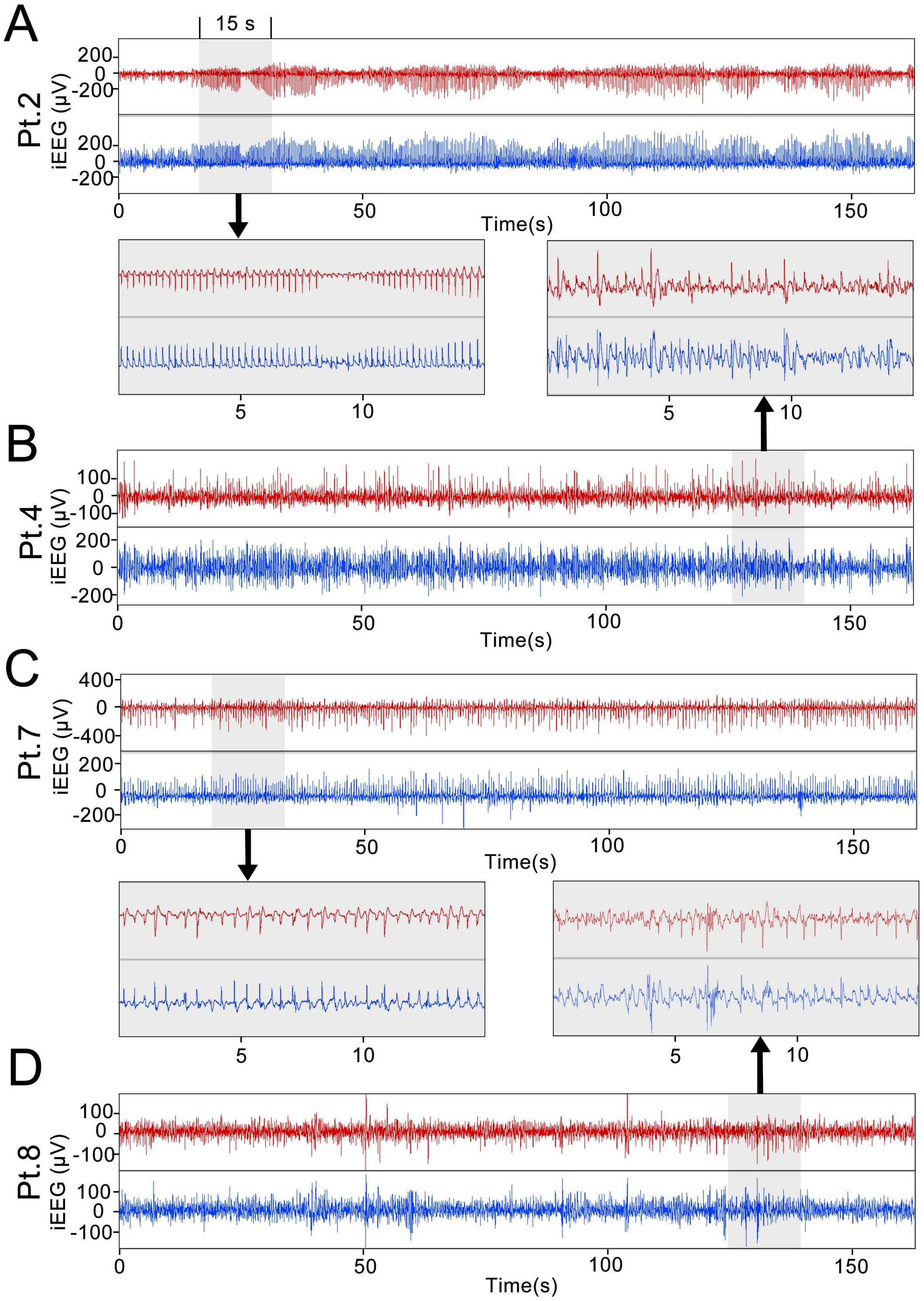

Supplementary Fig. 2: Interictal SEEG recordings from the EZ in four patients (Patients 2, 4, 7, and 8).

A-D Interictal EEG data (160s duration) from two pairs contacts within the EZ in four sample patients revealed synchronized, continuous and stable epileptiform discharges. Red and blue traces correspond to bipolar signals from two contact pairs within the EZ. Gray boxes highlight temporal segments: small boxes denote 15-s epochs, while large boxes indicate corresponding data displayed with an expanded time scale. Pt., patient; EZ, epileptogenic zone; iEEG, intracranial EEG.

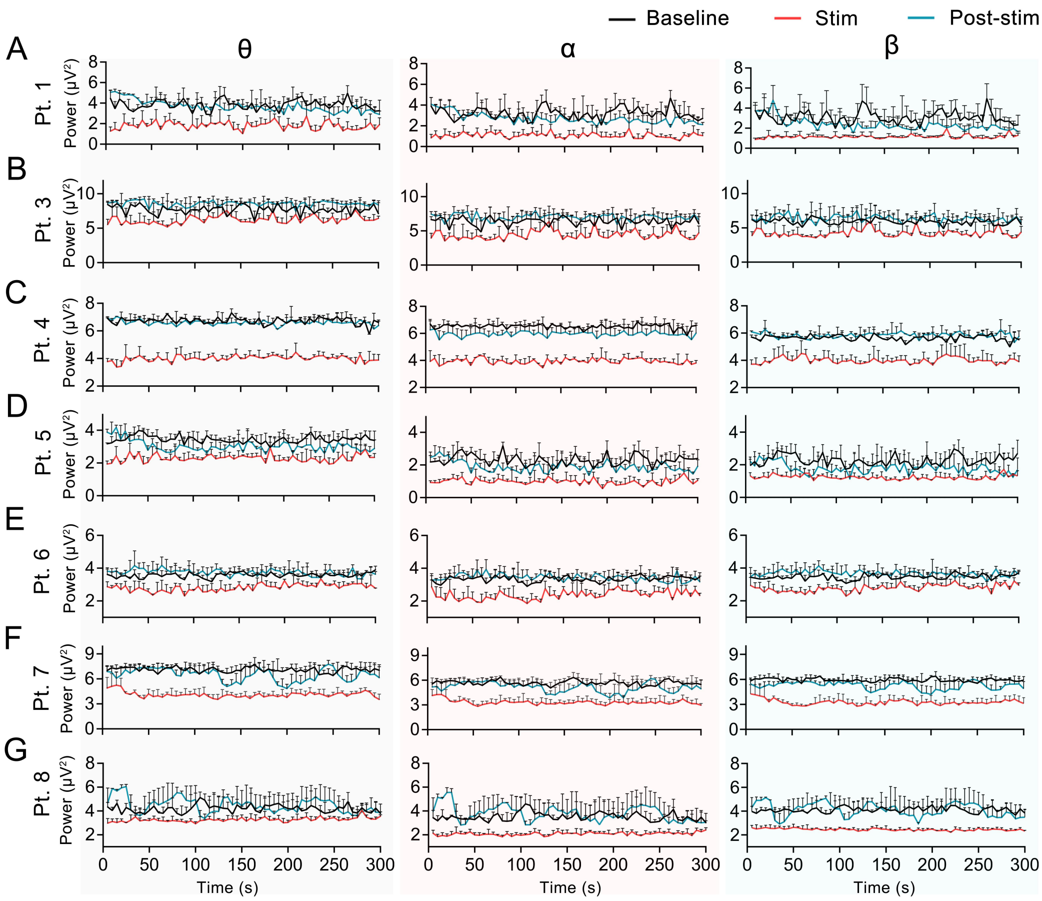

Supplementary Fig. 3 Power of the θ, α, and β bands in seven patients during the 5-min baseline, stimulation, and post-stimulation periods.

A-**G** The power in different frequency bands showed a stable decrease during the stimulation period, while the power of each frequency band fluctuated around baseline during the post-stimulation period. Data points reflect 5-s averaged power values in specific frequency band extracted from predefined 5-min epochs. Pt., patient.

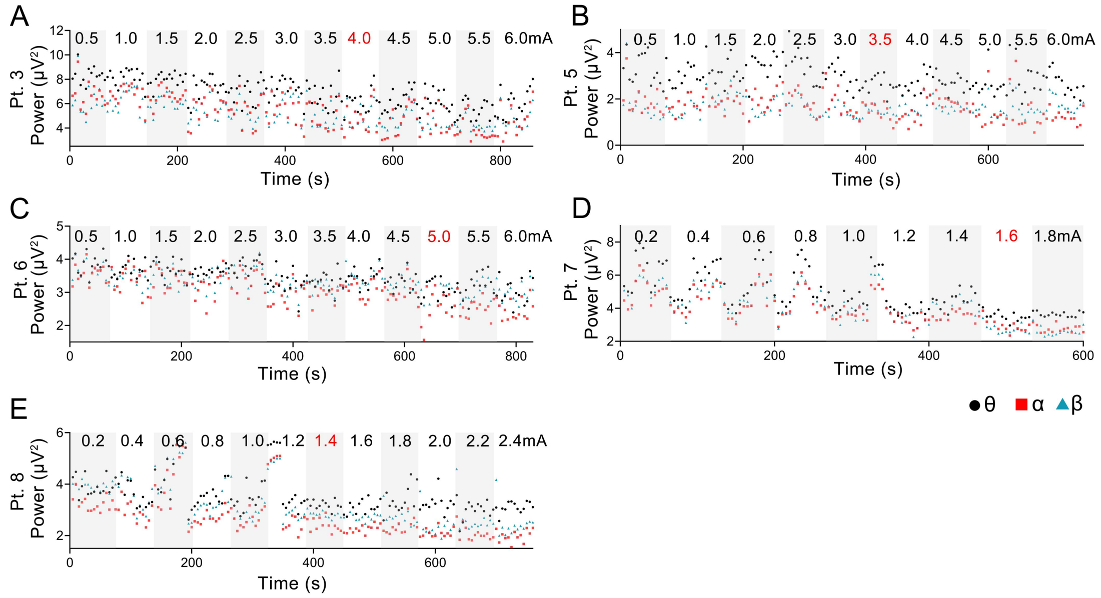

Supplementary Fig. 4 Power changes during incremental current stimulation in five patients.

A-E The power in different frequency bands gradually decreased during stimulation and stabilized after reaching a specific stimulation current (indicated by the red number). Data points represent 5-s averaged power values within specific frequency. Pt., patient.

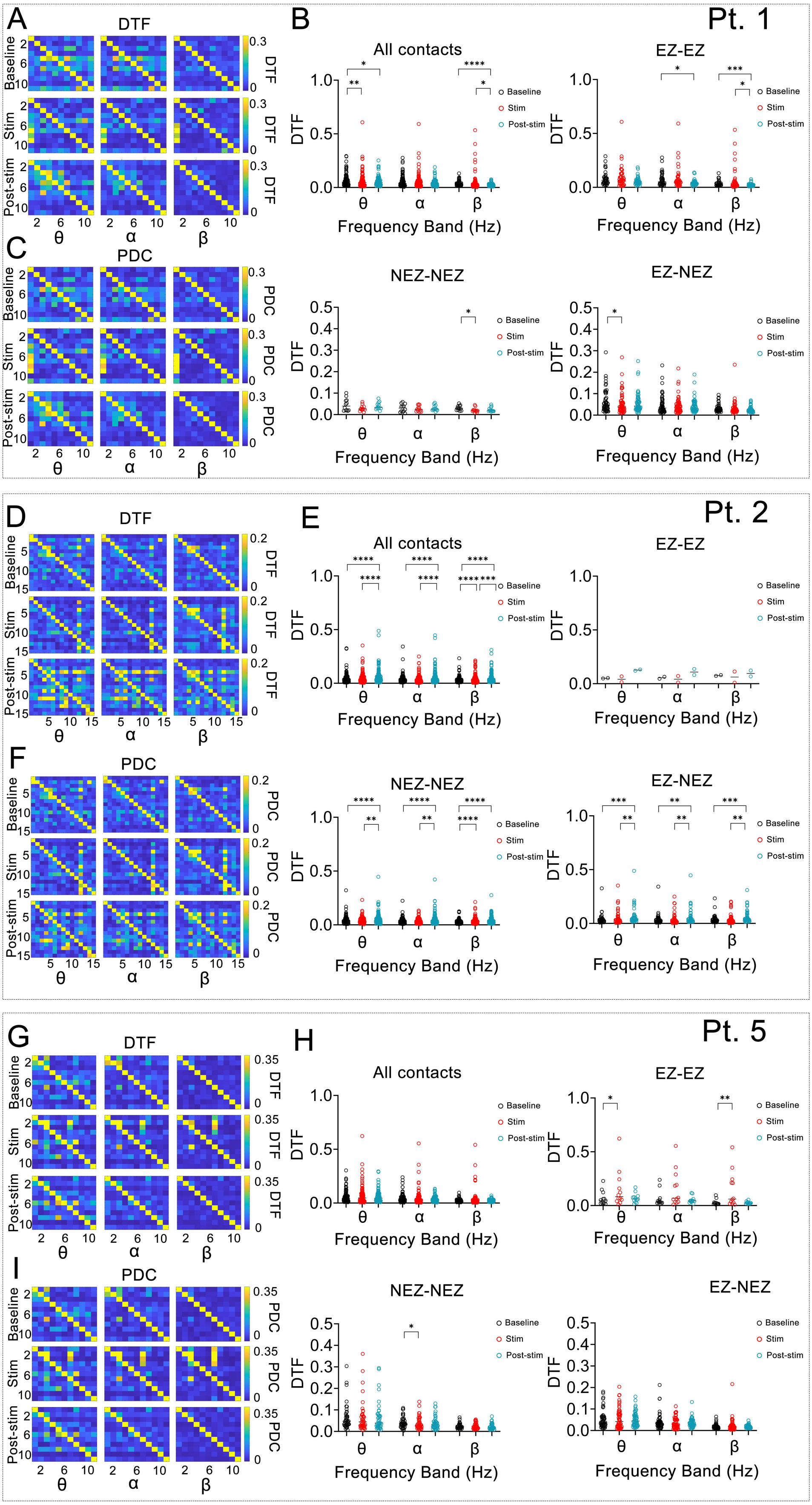

Pt. 1
A
DTF
Baseline
Stim
Post-stim
θ
α
β
B
All contacts
EZ-EZ
NEZ-NEZ
EZ-NEZ
Baseline
Stim
Post-stim
DTF
Frequency Band (Hz)
C
PDC
Pt. 2
D
E
F
Pt. 5
G
H
I

Supplementary Fig. 5 The effect of short-term high-frequency stimulation on the brain network in Patient 1, 2 and 5.

A, D and G Heatmaps of DTF values across different periods and frequency bands for Patients 1, 2, and 5, respectively. **C, F and I** Heatmaps of PDC values across different periods and frequency bands for Patients 1, 2, and 5, respectively; in panels A, C, D, F, G and I, the numerical labels on the x- and y-axes of each matrix represent the electrode contacts within selected brain regions (number of contacts: 11, 15, and 11 for Patient 1, 2, and 5, respectively). Each data point indicates the DTF or PDC value from the source (y-contact) to the target (x-contact) (n = 121, 225, and 121 for Patient 1, 2, and 5, respectively). **B, E and H** Comparisons of DTF between baseline, stimulation, and post-stimulation periods within specific frequency bands (theta, alpha, beta) and connection groupings (all contacts, EZ-EZ, NEZ-NEZ, EZ-NEZ). Parametric data (Shapiro test p-value ≥ 0.05) were analyzed with RM-ANOVA followed by Tukey's multiple comparisons test, whereas non-normally distributed datasets (Shapiro test p-value < 0.05) underwent Friedman tests followed by Dunn's multiple comparison test. (Patient 1, All contacts, n = 110, theta band: $\chi_F^2 = 12.02$, p = 0.0025; alpha band: $\chi_F^2 = 4.873$, p = 0.0875; beta band: $\chi_F^2 = 21.65$, p < 0.0001. EZ-EZ, n = 42, theta band: $\chi_F^2 = 5.905$, p = 0.0522; alpha band: $\chi_F^2 = 7.048$, p = 0.0295; beta band: $\chi_F^2 = 13.86$, p = 0.0010; NEZ-NEZ, n = 12, theta band: F= 2.813, p = 0.1091; alpha band: F = 0.2278, p = 0.7104; beta band: $\chi_F^2 = 8.167$, p = 0.0169. EZ-NEZ, n = 56, theta band: $\chi_F^2 = 8.143$, p = 0.0171; alpha band: $\chi_F^2 = 0.6786$, p = 0.7123; beta band: $\chi_F^2 = 5.607$, p = 0.0606. Patient 2, All contacts, n = 210, theta band: $\chi_F^2 = 44.34$, p < 0.0001; alpha band: $\chi_F^2 = 66.58$, p < 0.0001; beta band: $\chi_F^2 = 37.98$, p < 0.0001. NEZ-NEZ, n = 156, theta band: $\chi_F^2 = 25.42$, p < 0.0001; alpha band: $\chi_F^2 = 22.94$, p < 0.0001; beta band: $\chi_F^2 = 56.12$, p < 0.0001. EZ-NEZ, n = 52, theta band: $\chi_F^2 = 18.81$, p < 0.0001; alpha band: $\chi_F^2 = 15.35$, p = 0.0005; beta band: $\chi_F^2 = 16.65$, p = 0.0002. Patient 5, All contacts, n = 110, theta band: $\chi_F^2 = 0.3455$, p = 0.8414; alpha band: $\chi_F^2 = 5.582$, p = 0.0614; beta band: $\chi_F^2 =1.691$, p = 0.4294. EZ-EZ, n = 12, theta band: $\chi_F^2 = 7.167$, p = 0.0278; alpha band: $\chi_F^2 = 5.167$, p = 0.0755; beta band: $\chi_F^2 = 9.500$, p = 0.0087; NEZ-NEZ, n = 42, theta band: $\chi_F^2 = 6.048$, p = 0.0486; alpha band: $\chi_F^2 = 7.429$, p = 0.0244; beta band: $\chi_F^2 = 1.333$, p = 0.5134. EZ-NEZ, n = 56, theta band: $\chi_F^2 = 2.679$, p = 0.2620; alpha band: $\chi_F^2 = 4.750$, p = 0.0930; beta band: $\chi_F^2 = 3.250$, p = 0.1969.) (*p < 0.05, **p < 0.01, ***p < 0.001, ****p < 0.0001). Each data point corresponds to the mean DTF value calculated between paired electrode contacts within defined frequency bands and temporal intervals. Pt., patient; EZ, epileptogenic zone; NEZ, non-epileptogenic zone; DTF, directed transfer function; PDC, partial directed coherence.

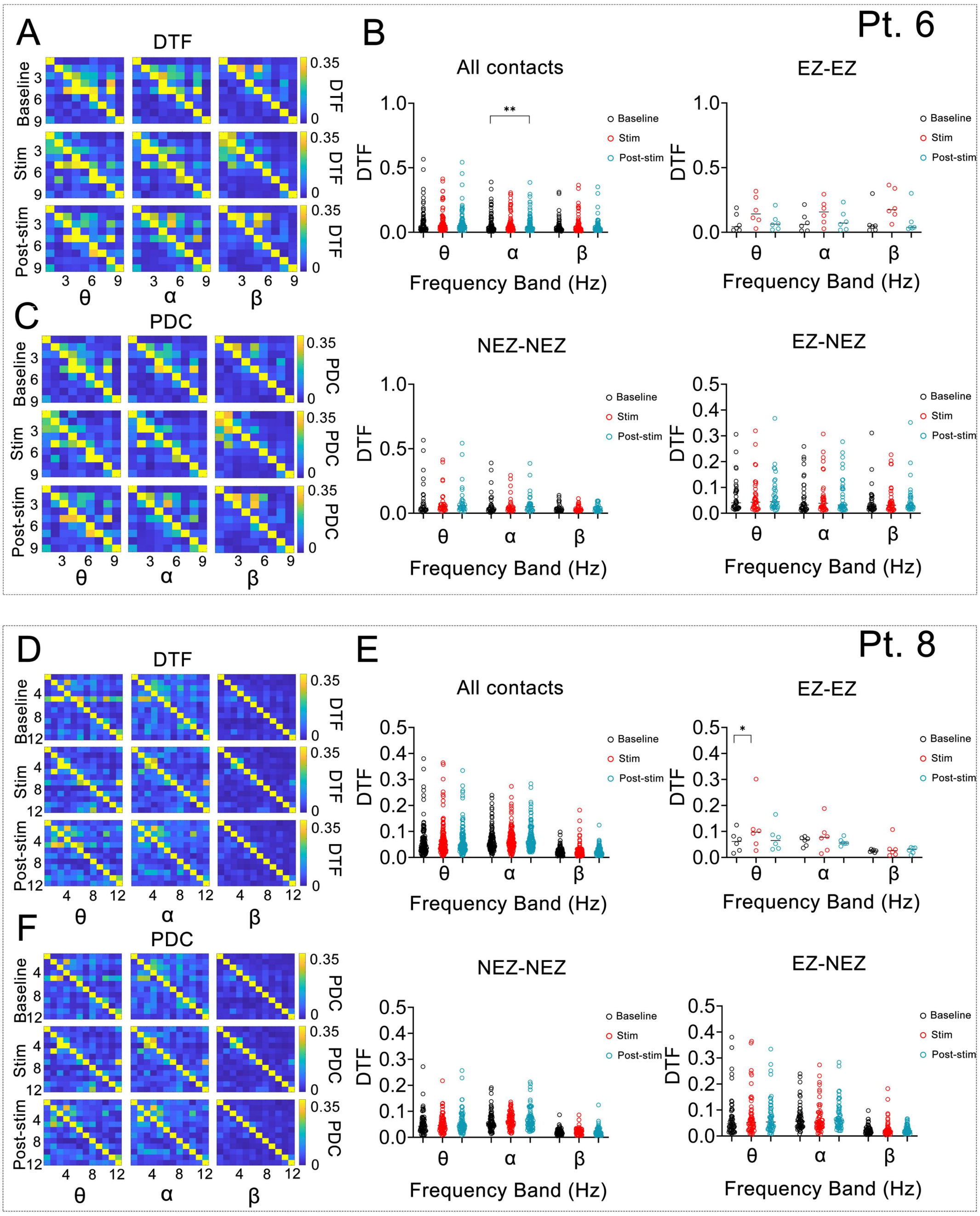

Supplementary Fig. 6 The effect of short-term high-frequency stimulation on the brain network in Patient 6 and 8.

A and D Heatmaps of DTF values across different periods and frequency bands for Patients 6 and 8, respectively. **C and F** Heatmaps of PDC values across different periods and frequency bands for Patients 6 and 8, respectively; in panels A, C, D and F, the numerical labels on the x- and y-axes of each matrix represent the electrode contacts within selected brain regions (number of contacts: 9 and 12 for Patient 6 and 8, respectively). Each data point indicates the DTF or PDC value from the source (y-contact) to the target (x-contact) (n = 81 and 144 for Patient 6 and 8, respectively). **B and E** Comparisons of DTF between baseline, stimulation, and post-stimulation periods within specific frequency bands (theta, alpha, beta) and connection groupings (all contacts, EZ-EZ, NEZ-NEZ, EZ-NEZ). Parametric data (Shapiro test p-value ≥ 0.05) were analyzed with RM-ANOVA followed by Tukey's multiple comparisons test, whereas non-normally distributed datasets (Shapiro test p-value < 0.05) underwent Friedman tests followed by Dunn's multiple comparison test (Patient 6, All contacts, n = 72, theta band: χ^2_F = 3.000, p = 0.2231; alpha band:

χ^2_F = 9.000, p = 0.0111; beta band: χ^2_F = 4.083, p = 0.1298. EZ-EZ, n = 6, theta band: F = 2.026, p = 0.2109; alpha band: F = 1.992, p = 0.2150; beta band: χ^2_F = 4.333, p = 0.1416; NEZ-NEZ, n = 30, theta band: χ^2_F = 3.267, p = 0.1953; alpha band: χ^2_F = 4.067, p = 0.1309; beta band: χ^2_F = 2.600, p = 0.2725. EZ-NEZ, n = 36, theta band: χ^2_F = 1.556, p = 0.4594; alpha band: χ^2_F = 5.389, p = 0.0676; beta band: χ^2_F = 2.389, p = 0.3029. Patient 8, All contacts, n = 132, theta band: χ^2_F = 4.288, p = 0.1172; alpha band: χ^2_F = 4.424, p = 0.1095; beta band: χ^2_F = 1.955, p = 0.3763. EZ-EZ, n = 6, theta band: χ^2_F = 7.000, p = 0.0289; alpha band: F = 0.6554, p = 0.4662; beta band: χ^2_F = 1.000, p = 0.7402; NEZ-NEZ, n = 72, theta band: χ^2_F = 2.861, p = 0.2392; alpha band: χ^2_F = 3.694, p = 0.1577; beta band: χ^2_F = 3.361, p = 0.1863. EZ-NEZ, n = 54, theta band: χ^2_F = 1.000, p = 0.6065; alpha band: χ^2_F = 1.815, p = 0.4036; beta band: χ^2_F = 0.2593, p = 0.8784.) (*p < 0.05, **p < 0.01, ***p < 0.001, ****p < 0.0001). Each data point corresponds to the mean DTF value calculated between paired contacts within defined frequency bands and temporal intervals. Pt., patient; EZ, epileptogenic zone; NEZ, non-epileptogenic zone; DTF, directed transfer function; PDC, partial directed coherence.

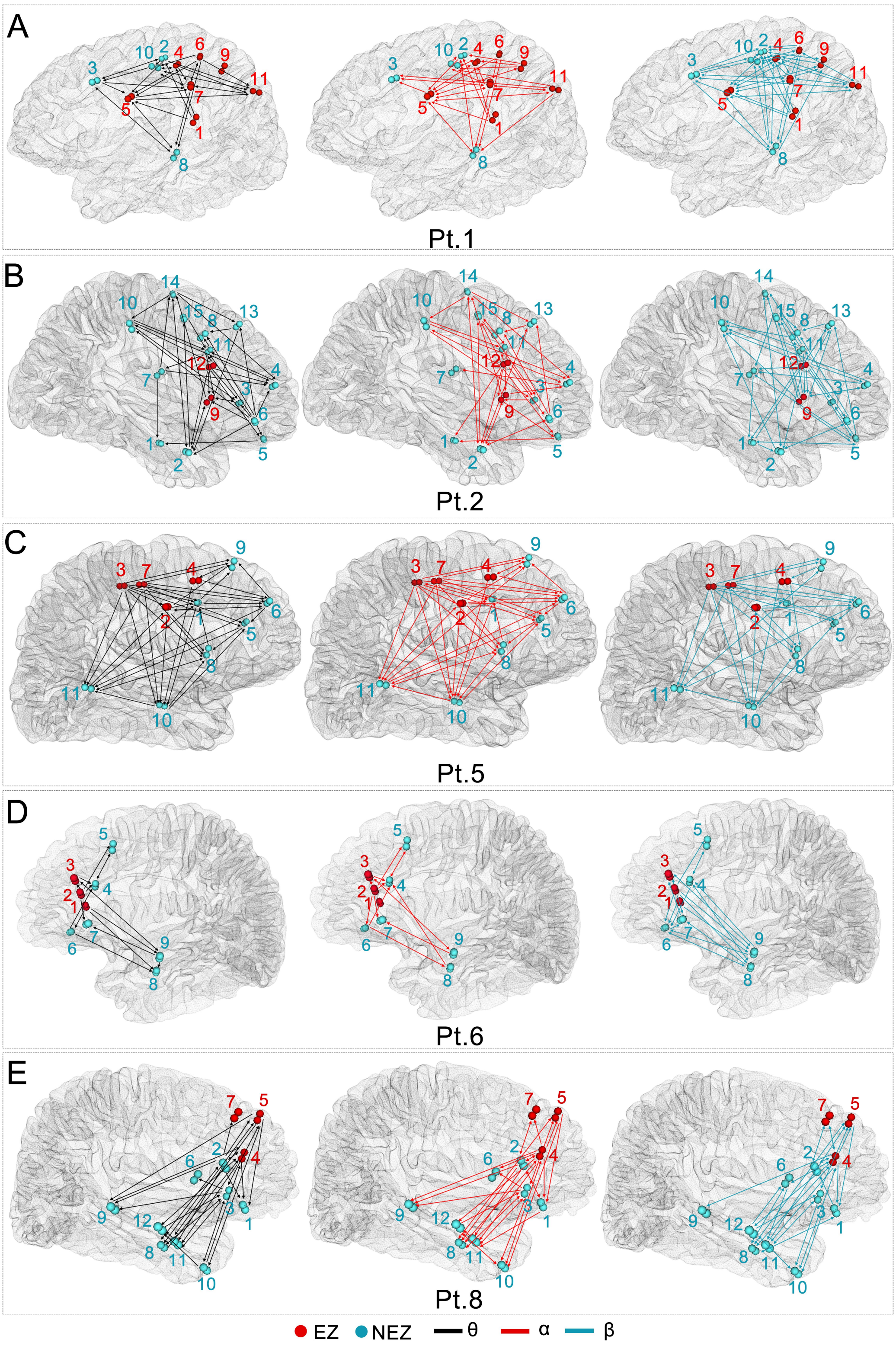

Supplementary Fig. 7 Stable frequency-specific suppression of the brain network during the three stimulation processes.

A-E Contact pairs in which PDC during the three stimulation processes across different frequency bands were lower than the average PDC during the baseline period. Red and blue contacts in the brain model denote the EZ and NEZ, respectively. Solid lines between contacts indicate connectivity, with arrowheads specifying the direction of PDC (from source to target contact). Pt., patient; EZ, epileptogenic zone; NEZ, non-epileptogenic zone; PDC, partial directed coherence.

Supplementary Tabel 1 Patient spike rate and amplitude in different period.

Pt.	Spike rate (/min) /Amp.(μV)	Baseline				Stimulation				Post-stimulation			
		block1	block2	block3	mean±SD	block1	block2	block3	mean±SD	block1	block2	block3	mean±SD
1	Rate	144.80	128.00	132.20	135±8.74	33.20	20.00	22.40	25.2±7.03	109.20	132.40	135.20	125.6±14.27
	Amp.	38.44	40.04	38.85	39.11±0.83	22.39	22.05	23.27	22.57±0.63	27.47	30.10	29.88	29.15±1.46
2	Rate	244.80	254.40	248.20	249.13±4.87	15.20	9.60	11.20	12±2.88	177.60	195.20	192.20	188.33±9.42
	Amp.	138.38	126.32	129.98	131.56±6.18	64.87	58.55	62.10	61.84±3.17	157.71	195.83	183.34	178.96±19.43
3	Rate	79.80	76.00	69.00	74.93±5.48	9.80	24.80	17.80	17.47±7.51	122.20	109.20	124.20	118.53±8.14
	Amp.	292.89	322.98	332.23	316.03±20.57	263.87	247.89	313.97	275.24±34.48	296.29	329.43	323.36	316.36±17.65
4	Rate	310.20	313.40	NA	311.8±2.26	16.40	11.00	NA	13.7±3.82	232.00	317.40	NA	274.7±60.39
	Amp.	99.90	105.33	NA	102.61±3.84	59.46	54.97	NA	57.21±3.17	113.91	106.95	NA	110.43±4.92
5	Rate	87.00	96.40	100.20	94.53±6.8	17.40	16.00	10.20	14.53±3.82	49.80	52.00	58.80	53.53±4.69
	Amp.	31.64	31.00	30.75	31.13±0.46	25.67	25.97	23.65	25.1±1.27	28.98	28.74	30.29	29.34±0.83
6	Rate	138.40	148.80	152.00	146.4±7.11	43.80	60.80	60.00	54.87±9.59	147.80	150.80	148.40	149±1.59
	Amp.	38.23	38.52	38.19	38.31±0.18	32.67	35.80	34.72	34.4±1.59	44.03	40.63	39.46	41.37±2.38
7	Rate	205.40	216.60	207.60	209.87±5.93	15.60	25.20	25.60	22.13±5.66	180.60	185.80	194.60	187±7.08
	Amp.	152.68	155.85	148.12	152.22±3.89	68.12	67.39	103.55	79.68±20.67	145.07	133.80	126.47	135.11±9.37
8	Rate	184.80	178.20	150.60	171.2±18.14	30.00	51.20	49.40	43.53±11.75	146.00	164.80	154.60	155.13±9.41
	Amp.	40.65	42.52	38.34	40.5±2.09	28.73	29.72	29.54	29.33±0.53	57.84	63.52	59.96	60.44±2.87

Amp., amplitude; Pt., patient; SD, standard deviation; NA, unrecorded

Supplementary Tabel 2 Median values of DTF changes during stimulation.

Pt.	group	ΔDTF θ	ΔDTF α	ΔDTF β
1	All contacts	-7.68×10^{-3}	-2.49×10^{-3}	-4.47×10^{-3}
	EZ-EZ	-7.52×10^{-3}	-6.19×10^{-3}	-5.07×10^{-3}
	NEZ-NEZ	-1.05×10^{-2}	-2.81×10^{-3}	-7.27×10^{-3}
	EZ-NEZ	-8.01×10^{-3}	-6.59×10^{-4}	-3.63×10^{-3}
2	All contacts	2.09×10^{-3}	1.56×10^{-3}	5.43×10^{-3}
	EZ-EZ	-9.02×10^{-3}	-1.37×10^{-2}	-1.47×10^{-2}
	NEZ-NEZ	1.80×10^{-3}	1.67×10^{-3}	6.73×10^{-3}
	EZ-NEZ	5.72×10^{-3}	-6.70×10^{-4}	6.73×10^{-4}
5	All contacts	-2.49×10^{-3}	-7.00×10^{-3}	1.47×10^{-3}
	EZ-EZ	2.58×10^{-2}	3.23×10^{-2}	4.02×10^{-2}
	NEZ-NEZ	-6.54×10^{-3}	-8.90×10^{-3}	-8.90×10^{-4}
	EZ-NEZ	-9.37×10^{-4}	-8.49×10^{-3}	1.10×10^{-3}
6	All contacts	-2.46×10^{-4}	1.76×10^{-3}	1.53×10^{-3}
	EZ-EZ	6.45×10^{-2}	9.24×10^{-2}	1.27×10^{-1}
	NEZ-NEZ	4.23×10^{-3}	3.25×10^{-5}	1.52×10^{-4}
	EZ-NEZ	-1.20×10^{-3}	3.17×10^{-3}	-3.58×10^{-4}
7	All contacts	-1.34×10^{-2}	-9.19×10^{-3}	-1.00×10^{-2}
	EZ-EZ	-2.03×10^{-1}	-1.38×10^{-1}	-1.27×10^{-1}
	NEZ-NEZ	-4.06×10^{-3}	-2.16×10^{-3}	-4.17×10^{-3}
	EZ-NEZ	-2.93×10^{-2}	-1.82×10^{-2}	-1.00×10^{-2}
8	All contacts	4.22×10^{-3}	9.12×10^{-4}	1.10×10^{-3}
	EZ-EZ	2.87×10^{-2}	1.15×10^{-2}	-6.98×10^{-4}
	NEZ-NEZ	2.36×10^{-3}	2.05×10^{-3}	1.86×10^{-3}
	EZ-NEZ	3.16×10^{-3}	-1.11×10^{-3}	-5.68×10^{-4}

EZ, epileptogenic zone; NEZ, non-epileptogenic zone; Pt., patient; ΔDTF, median values of directed transfer function changes (stimulation DTF – baseline DTF) in specific frequency bands during stimulation
